# Probiotic Characteristics and the Anti-Inflammatory Effects of *Lactiplantibacillus plantarum* Z22 Isolated from Naturally Fermented Vegetables

**DOI:** 10.3390/microorganisms12112159

**Published:** 2024-10-26

**Authors:** Shiyu Wang, Ziyu Nie, Li Zhu, Yanyang Wu, Yashi Wen, Fangming Deng, Lingyan Zhao

**Affiliations:** 1 College of Food Science and Technology, Hunan Agricultural University, Changsha 410128, China; 13462006093@163.com (S.W.); sry_zj@163.com (L.Z.); wuyanyang2002@126.com (Y.W.); wenyashi7758@163.com (Y.W.); 2 College of Animal Science and Technology, Hunan Biological Electromechanical Vocational College, Changsha 410128, China; 13467663507@163.com

**Keywords:** probiotic, anti-inflammatory activity, oxidative stress, NF-κB, whole genome analysis

## Abstract

Currently, there is increasing interest in the commercial utilization of probiotics isolated from traditional fermented food products. Therefore, this study aimed to investigate the probiotic potential of *Lactiplantibacillus plantarum* (*L. plantarum*) Z22 isolated from naturally fermented mustard. The results suggest that *L. plantarum* Z22 exhibits good adhesion ability, antibacterial activity, safety, and tolerance to acidic conditions and bile salts. We further determined the anti-inflammatory mechanism and properties of *L. plantarum* Z22 and found that *L. plantarum* Z22 could significantly reduce the secretion of pro-inflammatory cytokines, including interleukin-6 (IL-6), interleukin-1β (IL-1β), tumor necrosis factor-α (TNF-α), and the expression of the pro-inflammatory mediator cyclooxygenase-2 (COX-2) protein in LPS-induced RAW 264.7 cells. In addition, *L. plantarum* Z22 also effectively inhibited the signaling pathways of nuclear factor κB (NF-κB) and mitogen-activated protein kinases (MAPKs). This effect can be attributed to a decrease in the levels of reactive oxygen species (ROS) and increased heme oxygenase-1 (HO-1) expression. Moreover, whole-genome sequencing revealed that *L. plantarum* Z22 contains gene-encoding proteins with anti-inflammatory functions, such as beta-glucosidase (BGL) and pyruvate kinase (PK), as well as antioxidant functions, including thioredoxin reductase (TrxR), tyrosine-protein phosphatase, and ATP-dependent intracellular proteases ClpP. In summary, these results indicated that *L. plantarum* Z22 can serve as a potential candidate probiotic for use in fermented foods such as yogurt (starter cultures), providing a promising strategy for the development of functional foods to prevent chronic diseases.

## 1. Introduction

Probiotics, defined as “living microorganisms that, when administered in sufficient quantities, provide health benefits to the host” [1], are living cells with different beneficial properties. Lactic acid bacteria (LAB), commonly found in the intestines of healthy individuals, are well known for their immunomodulatory effects. In particular, certain strains belonging to the genus Lactobacillus may have health-promoting effects and are denoted as probiotics [2]. Several Lactobacillus species, such as *Lactobacillus plantarum*, *Lactobacillus. rhamnosus*, *Lactobacillus. Frumenti*, and *Lactobacillus. reuteri*, have been proven to have positive effects on the immunomodulation of the host. They can inhibit the development of pathogenic bacteria by improving the intestinal epithelial barrier functions and balancing the intestinal ecology [3]. In addition, a growing number of studies have shown that metabolites from intestinal flora may play a role in regulating inflammation-related diseases by influencing oxidative stress [4]. In recent years, there has been increasing interest in I confirm probiotic fermented foods, which has stimulated innovation and fueled the development of new products around the world. Research has revealed that strains originating from these environments display robust tolerance to acidic conditions and bile salts. In this case, these strains can survive in the human gastrointestinal tract at a pH of 2.5 and in the presence of 0.3% bile salt, offering benefits to the host through their metabolic activities [5]. Subrota et al. [6] found that *Limosilactobacillus fermentum* (*L. fermentum*) KGL4 and *Saccharomyces cerevisiae* (*S. cerevisiae*) WBS2A, isolated from fermented foods, exhibit strong probiotic properties and anti-inflammatory activity. Ambazagan et al. [7] also reported that five probiotics isolated from 14 kinds of Korean fermented foods exhibited antioxidant and antibacterial potential effects. Plenty of research has been carried out to isolate new probiotics (e.g., LAB) and explore their health-promoting potential. Considering LAB as the most important fermenting microorganisms with the ability to aid digestive health, probiotics (particularly LAB) isolated from fermented vegetables could be generally used as microbe-containing dietary supplements and can be considered an important functional food group [8]. In a previous study, Hyung-Seok Yu et al. [9] found that *Weissella cibaria* JW15 isolated from kimchi displayed anti-inflammatory potential. Thus, consuming probiotics is useful for maintaining health by protecting against pathogenic bacteria in the gut microbiota, and maintaining a normal balance of gut microbiota helps improve digestive health as well as the immune system [10]. Although fermented fruit and vegetables have been used as raw materials for probiotic microorganisms in several studies, there are still inadequate investigations of the probiotic potential of artisanal fermented vegetables [11]. Pickled potherb mustard is a widely consumed fermented vegetable product in China [12]. In China, the annual production of fermented mustard reaches 5.2 million tons, with a market value exceeding USD 1.2 billion [13]. In this context, this study successfully isolated *Lactiplantibacillus plantarum* (*L. plantarum*) Z22 from naturally fermented mustard and found that this strain has advantages such as easy cultivation. However, there has been relatively little research on probiotics isolated from fermented mustard. In contrast, the probiotics that have been studied more frequently are mainly derived from fermented meat products and yogurt. *L. plantarum* is the most important and prominent microorganism involved in the middle and latter steps of fermented vegetables, and its application and health potential merit further investigation [14]. Therefore, the isolation and identification of new strains is an important step to promote the research and development of probiotics.

Inflammatory responses and reactive oxygen species (ROS) play indispensable physiological functions in the immune defense system. However, the excessive or persistent production of ROS and inflammation can lead to many health problems. Inflammation is a complex response of the vascular tissues to harmful stimuli, such as pathogens, damaged cells, and irritants. It is mediated by a variety of signaling molecules produced by macrophages, monocytes, and mast cells [15]. Macrophages are among the most important innate immune cells that mediate inflammation through phagocytosis and the release of pro-inflammatory mediators [16]. They can be triggered by lipopolysaccharide (LPS) stimulation, leading to the activation of inflammatory cell signaling pathways, such as nuclear factor κB (NF-κB) and mitogen-activated protein kinases (MAPKs), including extracellular signal-related kinases (ERKs), c-Jun N-terminal kinases (JNKs), and p38 as the main kinases. This can result in phosphorylation which can lead to the production of pro-inflammatory mediators and cytokines such as nitric oxide (NO), prostaglandin E2 (PGE2), IL-1β, IL-6, and TNF-α [1]. It has been reported that the excessive release of pro-inflammatory cytokines can cause acute or chronic inflammatory diseases [17]. IL-6 could expand the inflammatory cascade, contributing to the inflammatory process, which plays an important role in the innate and adaptive immune response [18]. TNF-α, one of the primary and most potent pro-inflammatory cytokines, could promote the proliferation of several cells or could signal apoptosis, playing a central role in inflammation and immunity [19]. IL-1β also plays a vital role in the development of the inflammatory process and can lead to the secretion of other inflammatory cytokines, such as IL-6, IL-8, and TNF-α [20].

In addition, NF-κB, which is present in a variety of cells, is an important nuclear transcription factor in signaling pathways and is associated with inflammation [21]. Specifically, NF-κB plays a role in activating macrophages by inducing the production of cytokines [22]. MAPK signaling pathways participate in macrophage activation [23]. In response to inflammatory signals, the MAPK cascade is activated through the phosphorylation of p38, ERK, and JNK, with the activation of NF-κB [24]. Probiotics have been reported to possess anti-inflammatory effects by inhibiting the activation of MAPKs and NF-κB [25]. Nevertheless, even though several studies have demonstrated the beneficial effects of probiotics and their ability to modulate the immune system, thus providing anti-inflammatory activity, few studies describe the mechanisms of action of these microorganisms [19]. Therefore, all possible mechanisms and novel methods of inflammation reduction are of great interest.

The imbalance between oxidants and antioxidants results in oxidative stress. The human body has an antioxidant defense system, but when ROS production surpasses this capacity, excessive oxidation can lead to damage to cells or tissues [26]. A relationship between anti-inflammatory and antioxidant activities through the activation of the antioxidant enzyme HO-1 has been reported [27]. Recent studies have verified that ROS can induce oxidative stress, which activates inflammatory pathways, promotes macrophage polarization, and initiates cellular damage [28]. Moreover, the ROS-mediated activation of the MAPK signaling pathway could result in inflammatory cytokine production [29]. These reactions have been reported to be linked to the regulation of transcription factors such as NF-κB, which are crucial for the production of inflammatory proteins, including nitric oxide synthase (NOS) and cyclooxygenase (COX-2) [30]. Previous research has shown that probiotics have excellent antioxidant capacity, exerting these effects by producing various active cell surface components, proteins, and antioxidant enzymes. In this case, probiotics could prevent or hinder the progression of different oxidative-stress-related disorders [31].

Therefore, in this study, the probiotic potential of *L. plantarum* Z22 isolated from naturally fermented mustard was evaluated, including its acid resistance, bile salt tolerance, adhesion ability, and antimicrobial activity. In addition, the effects of the strain on pro-inflammatory cytokines, ROS production, COX-2, HO-1 proteins, and the related signaling pathways (NF-κB and MAPK) were investigated. Furthermore, whole-genome sequencing analysis was conducted on selected strains to further identify the relevant functional genes. We hope that this study will provide a theoretical basis regarding the development of functional probiotic products.

## 2. Materials and Methods

### 2.1. Materials and Reagents

The lactic acid bacteria used in this study belong to strain Z22. This strain was obtained from the plant-derived probiotic strain library at the Department of Food Science and Technology, Hunan Agricultural University, China. RAW264.7 (mouse macrophage) and HT-29 (human colorectal adenocarcinoma cell) were obtained from the National Collection of Authenticated Cell Cultures (Shanghai, China). LPS was purchased from Sigma (Shanghai, China). ELISA test kit was purchased from Lunchangshuo Biotechnology Co., Ltd. (Xiamen, China). H_2_O_2_ solution and DCFH-DA ROS fluorescent probe were purchased from Sopao Technology Co., Ltd. (Beijing, China). Bovine bile salt was bought from Huankai Microbial Technology Co., Ltd. (Guangdong, China). Hydrochloric acid was provided by Sinophosphoric Group Chemical Practice Co., Ltd. (Shanghai, China). Glyceraldehyde-3-phosphate dehydrogenase (GAPDH) was purchased from Cell Signaling Technology Co., Ltd. (Beverly, MA, USA). HO-1 primary antibody was purchased from Abcam biotechnology Co., Ltd. (Cambridge, UK). The primary antibodies for COX-2, IκB-α, p-IkB-α, p65, p-p65, ERK, p-ERK, JNK, p-JNK, p38, and p-p38 were obtained from Cell Signaling Technology (Beverly, MA, USA). Skim milk powder, RIPA buffer (strong), protease inhibitor mixture (100 × PIC), phosphatase inhibitor (10×), and ECL Plus supersensitive luminescent solution were purchased from Aibivitech Technology Co., Ltd. (Changsha, China), and BSA was bought from Saibao Co., Ltd. (Beijing, Chian). Developer and fixer were purchased from Jiaxin Co., Ltd. (Shanghai, China).

### 2.2. Bacterial Strain and Culture Conditions

The *L. plantarum* Z22 strains were isolated from traditional naturally fermented mustard in China and preserved at the Hunan Agricultural University’s (HUNAU) Key Laboratory Food Science (KLFS) in Hunan, China. *Escherichia coli* (*E. coli*, CGMCC 9181) was bought from CGMCCC (Beijing, China). *Staphylococcus aureus* (*S. aureus*, ATCC 6538) and *Salmonella enterica* subsp. *enterica* (*S. enterica*, ATCC 14028) were acquired from ATCC (Beijing, China). *E. coli*, *S. aureus*, and *S. enterica* were grown for 12 h in Nutrient Broth (NB, Guangdong Huankai Microbiology Technology Co., Ltd., Guangzhou, China) medium at 37 °C. *L. plantarum* Z22 was cultured for at least three consecutive generations using 2% (*v*/*v*) inoculum in each inoculation into the MRS broth before the experiments.

### 2.3. Cells Culture

RAW264.7 cells were maintained at 37 °C in the Roswell Park Memorial Institute (RPMI) 1640 medium containing 10% fetal bovine serum (FBS) and 1% antibiotics in a 5% CO_2_, while HT-29 cells were cultured in MCCOY’S 5A medium supplied with 10% FBS in a cell culture incubator of 5% CO_2_ at 37 °C. The medium was changed every 2–3 days.

### 2.4. Cell Viability

RAW264.7 cells (2 × 10^4^ cells/mL) were plated in 96-well plates for 24 h. The adherent cells were incubated with *L. plantarum* Z22 suspensions for 4 h at 37 °C (5% CO_2_). Then, cells were treated with 10% Cell Counting Kit-8 (CCK8) (Saint-Bio Co., Ltd., Shanghai, China) and incubated for another 2 h. The optical density (OD) was estimated at 450 nm using a microplate reader (Thermo Fisher Scientific, Waltham, MA, USA).

### 2.5. Acid Tolerance

The acid resistance of isolates was evaluated following the method described by Huang et al. [32], with some modifications. The strains were activated, centrifuged, washed twice with PBS (0.1 M, pH 7.2), and then re-suspended in PBS with pH 2.5 to a final concentration of 10^9^ CFU/mL. This was followed by the incubation at 37 °C for 0 h and 4 h. Live colonies A0 and A1 were counted on MRS agar, respectively. Each test was conducted in triplicate. The survival rates were determined based on the following formula:

(1) $$S\mathrm{urvival}\;\mathrm{rate}\left(\%\right)=\frac{A1}{A0}\times 100$$

where A0 represents the number of viable bacteria at 0 h and A1 represents the number of viable bacteria at 4 h.

### 2.6. Tolerance to Bile Salts

The strains were activated, centrifuged, washed twice with PBS (0.1 M, pH 7.2), and then re-suspended in PBS to a final concentration of 10^9^ CFU/mL. Subsequently, 1% of the cultures were transferred into MRS broth with 0.3% bovine bile salt in normal saline. Live colonies N0 and N1 were counted on MRS agar after 0 h and 2 h incubation at 37 °C [33]. Each test was conducted in triplicate. The survival rates were determined according to the following formula:

(2) $$\mathrm{Survival}\;\mathrm{rate}\left(\%\right)=\frac{N1}{N0}\times 100$$

where N0 represents the number of viable bacteria at 0 h, and N1 represents the number of viable bacteria at 2 h.

### 2.7. Hydrophobicity of the Cells Surface

The hydrophobicity of the cell surface was explored using microbial adhesion to hydrocarbons (MATH) method [34], with some modifications. The activated strain was centrifuged at 6000 rpm for 10 min. Then, the bacterial precipitate was collected, washed twice with PBS (0.1 M, pH 7.2), and re-suspended in 0.1 M KNO_3_ solution to a final concentration of 10^9^ CFU/mL. The OD at 600 nm of the suspension was adjusted to a value of 0.5 ± 0.02 (B0). After 1 mL of toluene was added to 3 mL of this suspension and vortex-mixed for 60 s, the mixture was kept at room temperature for 10 min to form two phases. The lower aqueous phase was carefully sucked and the corresponding absorbance (B1) was determined at 600 nm. Cell surface hydrophobicity was calculated using the following formula:

(3) $$\mathrm{MATH}\left(\%\right)=\frac{B0-B1}{B0}\times 100$$

where B0 represents bacterial suspension absorbance at 600 nm, and B1 represents aqueous phase absorbance at 600 nm.

### 2.8. Adhesion to HT-29 Cells

According to the method previously described by Ramos et al. [35], with some modifications, HT-29 cells (1 × 10^5^ cells/mL) were aliquoted into 6-well plates and incubated in 5% CO_2_ at 37 °C for 24 h. Then, the strain (1 × 10^9^ CFU/mL) was inoculated into HT-29 cells and incubated in a 5% CO_2_ atmosphere at 37 °C for 2 h. The bacterial suspension was sucked out, and HT-29 cells were then washed in PBS (0.1 M, pH 7.2) (five times). Then, 2 mL of methanol was added to each well for Gram staining. Microscopic examination was carried out under a 100 times oil microscope (Carl Zeiss Co., Ltd., Thuringia, Germany) and the number of bacteria adhered (C1) to HT-29 cells (C0) in 20 visual microscopic fields was counted. Each test was conducted in triplicate. The adhesion rates were calculated using the following formula:

(4) $$\mathrm{Adhesion}\;\mathrm{rate}\left(\%\right)=\frac{C1}{C0}\times 100$$

where C0 represents the number of HT-29 cells and C1 represents the number of bacteria.

### 2.9. Antimicrobial Activity

Three pathogenic strains, including *E. coli* (CGMCC 9181), *S. enterica* (ATCC 14028), and *S. aureus* (ATCC 6538), were exploited in this section. The antimicrobial activity in cell-free supernatant (CFS) was determined by the well diffusion method with slight modifications [36]. *Lactobacillus strain* was cultured in MRS broth at 37 °C for 18 h, centrifuged at 10,000 rpm for 10 min, and finally, the supernatant was filtrated through a sterile filter with a pore size of 0.22 μm. The pH of CFS was subsequently adjusted to 6.5. Mix the cultured pathogen liquid with the Luria-Bertani (LB) agar plates (Huankai Microbiology Technology Co., Ltd., Guangzhou, China) (the final concentration of the pathogenic bacteria is 1 × 10^6^ CFU/mL), then pour the mixture into Petri dishes. Make holes with a 6 mm diameter in each plate. A total of 100 µL of prepared supernatants was poured into wells and kept at 37 °C for 24 h and then the inhibition zones were measured.

### 2.10. Inflammatory Cytokines Assay

RAW264.7 cells were maintained in RPMI-1640 medium supplemented with 10% FBS at 37 °C in a humidified 5% CO_2_. The cells (confluency 80–90%) were detached using 1 mL of trypsin. The cells were then seeded in 48-well plates and washed 3 times after sticking to the wall. The bacterial suspension of live bacteria (10^10^ CFU/mL) and cells were treated in total for 4 h, and then lipopolysaccharide (LPS) solution with a concentration of 2.5 μg/mL was added. After 12 h of stimulation, the supernatant was collected by 1000 rpm centrifuge (BOECO Co., Ltd., Osterode am Harz, Germany). The levels of IL-1β, IL-6, and TNF-α in the supernatant were determined using an ELISA kit (Lunchangshuo Biotechnology Co., Ltd., Xiamen, China) [37].

### 2.11. Measurement of ROS

RAW264.7 cells (2 × 10^4^ cell/well) were seeded into 96-well plates and incubated in 5% CO_2_ at 37 °C for 24 h. The bacterial suspension of live bacteria (10^10^ CFU/mL) was inoculated into RAW264.7 cells, followed by incubation in a 5% CO_2_ atmosphere at 37 °C for 2 h. After incubation, these cells were treated with an LPS solution (2.5 μg/mL) and further incubated for 12 h. Finally, these cells were cultured at 37 °C in the dark for 20 min in RPMI 1640 medium supplemented with 10 μM 2′,7′-Dichlorodihydrofluorescein diacetate (DCFHDA) (Solarbio Co., Ltd., Beijing, China). Fluorescence was read using a fluorescence microplate reader (Thermo Fisher Scientific, Waltham, MA, USA) at excitation/emission wavelengths of 485/530 nm [38]. Relative ROS levels were calculated using the following formula:

(5) $$\mathrm{ROS}\left(\%\right)=\frac{{\mathrm{OD}}_{\mathrm{sample}}}{{\mathrm{OD}}_{\mathrm{control}}}\times 100$$

where OD_sample_ represents sample absorbance and OD_control_ represents control absorbance.

### 2.12. Western Blot

RAW264.7 cells were cultured with 10^10^ CFU/mL live bacteria and LPS in 6-well culture plates for 18 h, and the cells were collected after lysis. Protein loading buffers were added to these collected materials and incubated in a metal bath (100 °C) for 10 min. The same amount of protein from each treatment group (30 μg) was separated on 10% SDS-polyacrylamide gel electrophoresis (SDS-PAGE) and transferred onto polyvinylidene difluoride (PVDF) membranes. The transferred immunoblots were blocked with 5% skim milk, and then the membrane was probed with the appropriate primary antibodies against HO-1, COX-2, IκB-α, p-IkB-α, p65, p-p65, ERK, p-ERK, JNK, p-JNK, p38 and p-p38 at 4 °C overnight. After brief washing with PBST, the transferred immunoblots were incubated with secondary antibodies at room temperature for 1 h. This was followed by the subsequent washing with PBST. Then, these blots were detected on an X-ray blue film using an enhanced chemiluminescence reagent.

### 2.13. Whole Genome Sequencing

Whole genome sequencing was achieved as described by Li et al. [39]. Strain Z22 genomic DNA was extracted using the DNeasy Tissue kit (Merck, Darmstadt, Germany) following the manufacturer’s instructions and submitted to Major Biomedical Technology Co., Ltd. (Shanghai, China) for whole genome sequencing using the Pacific Biosciences Sequel platform and the Illumina HiSeq platform (PE150 mode).

### 2.14. Statistical Analysis

The data was analyzed using SPSS 27.0 software (IBM, New York, NY, USA). The results were analyzed statistically and represented as mean ± SD. Statistical significance was calculated using One-way ANOVA followed by Tukey’s post hoc test. *p* < 0.05 was considered statistically significant.

## 3. Results and Discussion

### 3.1. Insight into Probiotic Potential of L. plantarum Z22

#### 3.1.1. Acid and Bile Tolerance

The ingested probiotics must survive passage through the human gastrointestinal tract to function properly [40]. Consequently, the ability to resist acid and bile salts is a crucial trait for probiotics. To explore the acid and bile tolerance of *L. plantarum* Z22, its tolerance to the conditions of pH 2.5 and 0.3% bile salts was tested (as described in Section 2.5 and Section 2.6) and the results are presented in Table 1. As shown in Table 1, after incubation at pH 2.5 for 4 h, the strain continued to grow with an increased viable count, and the survival rate reached 102.20%. Moreover, the survival rate of the strain reached 101.19% after incubation with 0.3% bile salt for 2 h. It can be seen that the strain is resistant to bile salts of 0.3% and pH of 2.5. There were reports that tolerance to gastric acidity (pH 2.0–2.5) is considered a key functional requirement for probiotics, enabling them to survive during passage through the gastrointestinal tract [41]. Gilliland et al. [42] discovered that the bile salt concentration in the human small intestine can reach up to 0.3% (*w*/*v*). Similarly, Lee and Park. [43] reported that *L. plantarum* isolated from kimchi, a traditional Korean fermented cabbage, showed reasonable survivability after 3 h of exposure to pH 3.0 and 0.3% bile salts. Therefore, it can be concluded that the strain exhibited high viability in the gastrointestinal environment.

#### 3.1.2. Hydrophobicity and Adhesion Abilities

Hydrophobicity and adhesion are considered important selection criteria for potential probiotic strains [44]. The ability to adhere to the intestinal epithelium and colonize is another important feature for potential probiotic candidates. The health benefits of probiotics are linked to the composition of gut microbiota and their ability to adhere to epithelial and mucosal surfaces [26]. Thus, an important parameter for the functional properties of probiotics is their ability to adhere to the intestine. In this study, the hydrophobic properties of *L. plantarum* Z22 and its adhesion to HT-29 cells were determined as described in Section 2.7 and Section 2.8. The results are shown in Table 2, where it can be observed that the hydrophobicity and the adhesion of strain Z22 reached 45.67 ± 3.06% and 72.75% ± 16.69, respectively. Studies have shown that, when the hydrophobicity of the strain is 40~60%, it is considered to be moderately hydrophobic [45]. Previously, Jacobsen et al. [46] divided the bacterial adhesion performance into three categories: (1) adhesion of less than 40 bacteria: non-adhesion, (2) adhesion of 41–100 bacteria: moderate adhesion, and (3) adhesion of more than 100: strong adhesion. Therefore, *L. plantarum* Z22 can be regarded as moderately hydrophobic with moderate adhesion. These results are similar to those reported by Ahmad [47], who also found that *Lactobacillus plantarum* isolated from fermented durian (tempoyak) showed a strong adhesive capacity (159 ± 10) in the human intestinal cell line HT-29. The adhesion of bacterial cells is usually related to their surface characteristics, such as pili, adhesins, mucus-binding proteins, fibronectin-binding proteins (FBPs), surface-layer proteins (SLPs), lipoteichoic acid, and exopolysaccharides [48,49,50]. However, the adhesion of probiotic strains varies among strains, depending on the surface characteristics of the cells [51]. Thus, it has been proven that this strain can colonize the intestine and maintain the intestinal microflora homeostasis of the host.

#### 3.1.3. Antimicrobial Activity

Antimicrobial activity is an important property for assessing probiotic potential [52]. To further understand the probiotic potential of *L. plantarum* Z22, its antimicrobial activity toward Gram-positive bacteria (*S. aureus*) and Gram-negative bacteria (*E. coli* and *S. enterica*) was evaluated. The results are shown in Table 3, indicating that the supernatant exhibited the most potent inhibitory effect on *S. aureus*, with the zone reaching a diameter of 20.67 ± 0.03 mm, while the supernatant displayed the lowest inhibitory activity against *S. enterica*, with an inhibition zone of 19.16 ± 0.04 mm. These results indicate that the strain (in the form of neutral cell-free supernatant) could inhibit the growth of indicator bacteria, but showed different extents of antimicrobial activities to different bacteria. In addition, it can be seen that the inhibitory zone diameter of *S. aureus* (Gram-positive bacteria) was significantly larger than that of *E. coli* and *S. enterica* (Gram-negative bacteria). This might suggest *L. plantarum* Z22 showed higher antimicrobial activities against Gram-positive bacteria than Gram-negative bacteria.

### 3.2. Understanding the Anti-Inflammatory Mechanism of L. plantarum Z22

#### 3.2.1. Inhibition of Pro-Inflammatory Cytokines Release in RAW264.7 Cells by *L. plantarum* Z22

**Figure 1:**
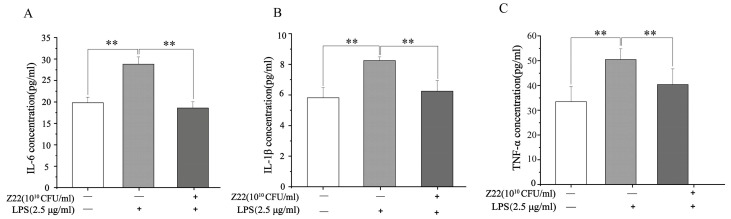
*L. plantarum* Z22 inhibits proinflammatory cytokine (IL-6, IL-1β, and TNF-α) secretion in LPS-induced RAW264.7 cells. The levels of IL-6 (**A**), IL-1β (**B**), and TNF-α (**C**) in supernatants of RAW264.7 cell culture (pretreated with the strain suspensions for 4 h and then stimulated with LPS for 12 h). Each group was repeated at least three times. Error bars, S.D.** means *p* < 0.01 compared to control group by one-way ANOVA.

In a pre-test, we found that *L. plantarum* Z22 had no toxic effect on RAW264.7 cells in a CCK8 assay, so this strain can be used with RAW264.7 cells. In this study, to determine the anti-inflammatory effects of *L. plantarum* Z22, the production of IL-6, IL-1β, and TNF-α was measured in LPS-induced RAW264.7 cells, and the related data are presented in Figure 1. Figure 1 exhibits the inhibitory effect of *L. plantarum* Z22 on the release of pro-inflammatory factors in RAW264.7 cells. As shown in Figure 1A–C, the IL-6 (28.82 pg/mL), IL-1β (8.24 pg/mL), and TNF-α (50.34 pg/mL) concentrations in the LPS group were significantly higher than in the control group, implying that LPS treatment could increase the pro-inflammatory secretion of cytokines. However, after *L. plantarum* Z22 treatment, the levels of IL-6 (18.53 pg/mL), IL-1β (6.25 pg/mL), and TNF-α (40.37 pg/mL) were significantly reduced. Inflammation is a primary host defense mechanism against many stimuli [16]. IL-1β, IL-6, and TNF-α are important inflammatory cytokines that are secreted by activated macrophages and are involved in promoting various inflammatory responses and pathological pain [53]. Studies have shown that probiotic bacteria lead to an increase in immune cell proliferation and diminish the production of pro-inflammatory cytokines [54]. In recent years, probiotics have been shown to be effective in treating inflammation. Our findings concur with those of Chon et al. [24], who reported that *L. plantarum* 10hk2 isolated from fermented vegetables could reduce IL-1β, IL-6, and TNF-α levels in LPS-induced RAW264.7 cells and thus exert anti-inflammatory effects. In this study, we found that *L. plantarum* Z22 can significantly reduce the levels of IL-6, IL-1β, and TNF-α. Therefore, it may play a role in inflammatory diseases by influencing the production of anti-inflammatory factors for anti-inflammatory purposes.

#### 3.2.2. Inhibition of NF-κB Activation and IκB-α Phosphorylation in RAW264.7 Cells by *L. plantarum* Z22

**Figure 2:**
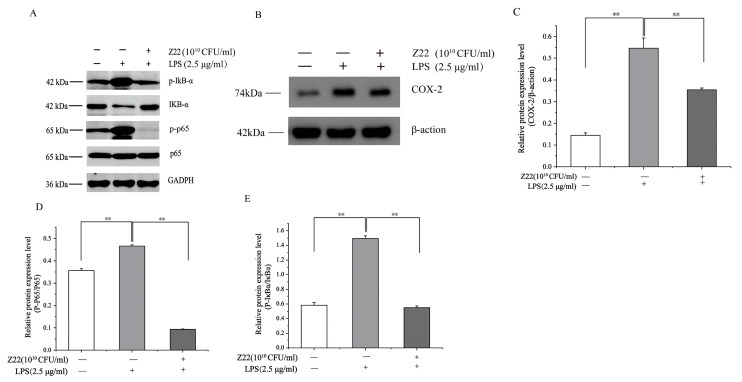
*L. plantarum Z22* inhibits NF-κB activation and COX-2 expression in LPS-induced RAW 264.7 cells. (**A**) The protein expression (tested by the method of Western blotting) of p-IκB-α, IkB-α, p65, and p-p65 in cells treated with *L. plantarum* Z22 for 18 h. (**B**) The protein expression (tested by the method of Western blotting) of COX-2 from the cells treated with *L. plantarum* Z22 for 18 h. (**C**–**E**) Band densities were measured using Image J 1.54 software and subjected to statistical analysis. Note: Blank control: Untreated cells, Model group: The cells were treated with LPS only, Treatment group: The cells were treated with *L. plantarum* Z22 and LPS. Each group was repeated at least three times. ** Significant differences between the groups, *p* < 0.01.

NF-κB is a major transcription factor that regulates the expression of pro-inflammatory genes. As an important downstream pathway of the LPS-mediated signaling response, NF-κB is closely related to tumor growth, inflammation, and apoptosis [55]. When macrophages are stimulated, the inactive IκBα and p65 complexes linked to Iκbα in the cytoplasm are ubiquitinated and degraded, and the active p65 is released into the nucleus, where it binds to pro-inflammatory genes, resulting in an inflammatory response [56]. Therefore, the expression of IκBα protein and the active form of p65 transcription are important markers of inflammatory response. Thus, to further explore the signaling mechanisms associated with the anti-inflammatory effects of *L. plantarum* Z22, the effect of *L. plantarum* Z22 on the activation of NF-κB was investigated in LPS-induced RAW264.7 cells. As shown in Figure 2A,D,E, compared to the blank control, LPS induction exhibited a significant increase in p-IκBα and p-p65 levels in the RAW264.7 cells. However, *L. plantarum* Z22 reduced the expression of p-IκBα and p-p65. Wang et al. [57] also found that Lactobacillus acidophilus (*L. acidophilus*) played an anti-inflammatory and protective role by downregulating the expression of p-IκBα protein, which is consistent with our results. COX-2 is an inflammatory factor commonly regarded as a downstream modulator of NF-κB and can cause inflammatory damage to cells. To determine whether COX-2 is implicated in the anti-inflammatory effect of *L. plantarum* Z22, the level of COX-2 expression was examined in LPS-stimulated RAW264.7 cells pre-treated with *L. plantarum* Z22. As shown in Figure 2B,C, the levels of COX-2 protein were upregulated by LPS stimulation and were downregulated following treatment with *L. plantarum* Z22, so the treatment of *L. plantarum* Z22 inhibited the expression of COX-2. Hao et al. [58] found that *L. plantarum* T1, isolated from pickled vegetables, significantly reduced the expression of COX-2 induced by LPS-induced RAW264.7 cells at the protein and also activated the NF-κB pathway. Thus, these results indicate that *L. plantarum* Z22 inhibits the inflammatory reaction through modulating the NF-κB signaling pathway and COX-2 expression.

#### 3.2.3. Inhibition of MAPK Activation in RAW264.7 Cells by *L. plantarum* Z22

**Figure 3:**
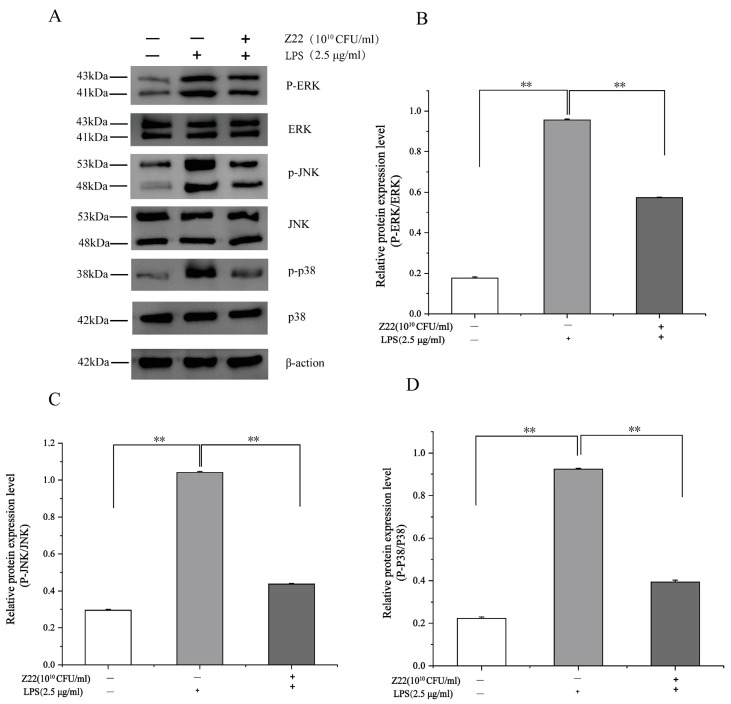
*L. plantarum* Z22 inhibits MAPK activation in RAW264.7 cells induced by LPS. (**A**) The protein expression (tested by the method of Western blotting) of p38, p-p38, JNK, p-JNK, ERK, and p-ERK from the cells treated with *L. plantarum* Z22 for 18 h. (**B**–**D**) Band densities were measured using Image J software and subjected to statistical analysis. Note: Blank control: Untreated cells, Model group: The cells were treated with LPS only, Treatment group: The cells were treated with *L. plantarum* Z22 and LPS. Each group was repeated at least three times. ** Significant differences between the groups, *p* < 0.01.

The MAPK signaling pathways are crucial for cell proliferation, differentiation, and activation, and include three primary members, namely p38, JNK, and ERK, which regulate gene expression [59]. The MAPK signaling pathway can induce the transcriptional activation of NF-κB through p38, JNK, and ERK phosphorylation [60]. In this part of the study, to gain more insight into the anti-inflammatory properties of *L. plantarum* Z22, the effect of *L. plantarum* Z22 on MAPK activation was determined in RAW264.7 cells stimulated by LPS. Compared to the blank control, after exposure to LPS, the levels of phosphorylated p38, ERK, and JNK noticeably increased in the RAW264.7 cells compared with non-treated cells (Figure 3). However, treatment with *L. plantarum* Z22 suppressed the phosphorylation of MAPKs. Lee et al.’s [61] research found that CFS obtained from selected LAB strains effectively stimulated the phosphorylation of MAPKs, including ERK, JNK, and p38, in RAW264.7 cells. This finding is similar to our results. In addition, it has been reported that numerous strains of *L. plantarum* possess anti-inflammatory effects [62]. Fermented mustard greens are widely used in China; however, there is little research on the Lactobacillus strains isolated from them. Our results showed that *L. plantarum* Z22 suppresses LPS-induced inflammation through the inhibition of MAPK activation and NF-κB translocation, demonstrating good anti-inflammatory capabilities. This suggests that *L. plantarum* Z22 may become an effective natural anti-inflammatory agent, providing new ideas and directions for the prevention and treatment of related inflammatory diseases.

### 3.3. L. plantarum Z22 Inhibited ROS Production and Increased HO-1 Expression in LPS-Induced RAW264.7 Cells

**Figure 4:**
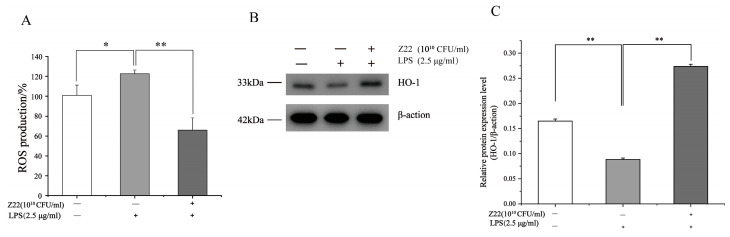
*L. plantarum* Z22 inhibited ROS production and increased HO-1 expression in LPS-induced RAW264.7 cells. (**A**) RAW264.7 cells were pretreated with the strain suspensions for 2 h and then stimulated with LPS for 12 h. The fluorescence level of ROS was measured by DCFHDA. (**B**) The protein expression (tested by the method of Western blotting) of HO-1 from the cells treated with *L. plantarum* Z22 for 18 h. (**C**) Band densities were measured using Image J software and subjected to statistical analysis. Note: Blank control: Untreated cells, Model group: The cells were treated with LPS only, Treatment group: The cells were treated with *L. plantarum* Z22 and LPS. Each group was repeated at least three times. Error bars, s.d. * means *p* < 0.05 compared to control group by one-way ANOVA. ** means *p* < 0.01 compared to control group by one-way ANOVA.

ROS are byproducts of aerobic metabolism and can induce a series of inflammatory responses when overexpressed. HO-1 is a multifunctional regulator of oxidative-stress-related diseases, which can protect cells from oxidative damage. However, a high expression of HO-1 inhibits LPS-induced ROS production, thereby preventing oxidative damage in cells, regulating inflammation, and regulating apoptosis. To determine the antioxidant activity, ROS production and HO-1 protein expression were measured in LPS-induced RAW264.7 cells. As shown in Figure 4A, treatment with LPS significantly increased the production of ROS (100.82%), in contrast to the control (122.60%). Nevertheless, pre-treatment with *L. plantarum* Z22 inhibited ROS production to 65.99%. Meanwhile, the treatment of this strain also increased the expression of HO-1 (Figure 4B,C). Some research has reported that the high expression of HO-1 inhibited LPS-induced ROS production, leading to a reduction in pro-inflammatory cytokine levels in RAW264.7 mouse macrophages [63]. Thus, the results from this study might indicate that *L. plantarum* Z22 has antioxidant capacity.

### 3.4. Phylogenetic Tree Construction and Genome Properties of L. plantarum Z22

**Figure 5:**
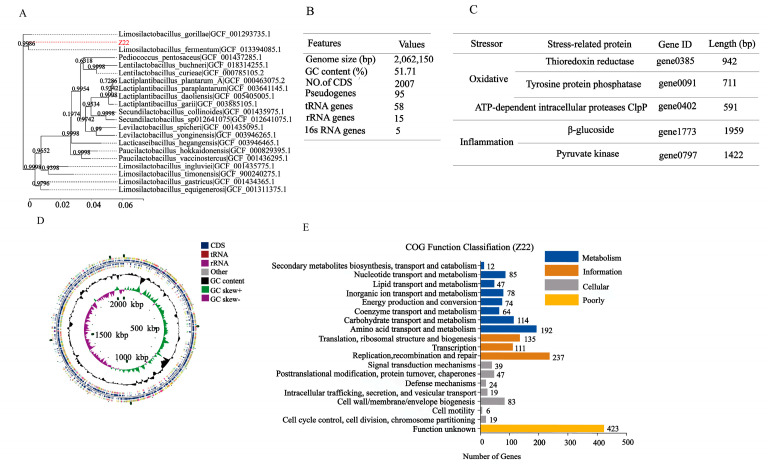
Genome features of *L. plantarum* Z22. (**A**) Phylogenetic tree of *L. plantarum* Z22 based on 16S rRNA genes. (**B**) General genomic features of *L. plantarum* Z22. (**C**) Representative related to the anti-inflammatory oxidation genes of *L. plantarum* Z22. (**D**) Circular genome map of *L. plantarum* Z22. From the outer circle to the inner, information is displayed as follows: Forward strand CDS, Reverse strand CDS, tRNA genes, tRNA genes, GC content, GC skew, and Genome size. (**E**) COG Functional annotation of *L. plantarum* Z22. Different bar colors represent the further classification of all functional categories into four major classes—Poorly Characterized (yellow bars), Cellular Processes and Signaling (gray bars), Information Storage and Processing (orange bars), and Metabolism (blue bars).

To obtain functional information, the whole genome sequencing of *L. plantarum* Z22 was analyzed and the corresponding results are shown in Figure 5. Figure 5A presents the Phylogenetic tree of *L. plantarum* Z22 based on 16S rRNA genes. When comparing the 16S RNA sequence of strain Z22 with the NCBI gene bank, it can be seen that strain Z22 had 100.00% homology with *Lactiplantibacillus plantarum*, and was identified as *Lactiplantibacillus plantarum*. Whole-genome sequencing and comprehensive bioinformatic analysis were employed for the investigation of the genomic features of strain Z22 (Figure 5B). Based on the genomic features, the corresponding genome map can be constructed (Figure 5D). Figure 5B suggests that the Z22 strain has a single, circular chromosome, 0 plasmids, a genome length of 2,062,150 bp, and a GC content of 51.71%. Among the predicted genes, 2007 were found to be protein-coding sequences (CDSs). Furthermore, 95 pseudogenes, 58 tRNAs, 15 rRNAs, and 5 16S rRNA genes were identified (Figure 5B). To evaluate the safety of this strain, it is necessary to analyze the genes associated with antibiotic resistance in *L. plantarum* Z22. However, according to ResFinder_v4.1.0 databases (https://cge.cbs.dtu.dk/services/ResFinder/, accessed on 25 November 2022), no antibiotic resistance genes were identified in *L. plantarum* Z22. In addition, the presence of mobile genetic elements and transposons was investigated. The results demonstrated that *L. plantarum* Z22 possesses 23 transposons, while no plasmids were observed.

To further explore the functional characteristics of the genomes, COG classification was performed and data are shown in Figure 5E. Based on the COG database, 1780 protein-coding genes were assigned to families comprising 19 functional categories into four types: cellular, metabolism, information, and function unknown. The COG classification indicates that *L. plantarum* Z22 was involved in the following aspects: (1) translation/ribosomal structure and biogenesis, (2) amino acid transport and metabolism, (3) carbohydrate transport and metabolism, (4) energy production and conversion, (5) coenzyme transport and metabolism, and (6) secondary metabolite biosynthesis, transport, and catabolism. In addition, we also found some anti-inflammatory genes, such as beta-glucosidase and pyruvate kinase (PK), in the genome (Figure 5C). It has been reported that beta-glucosidase (BGL) from plants can protect against cardiovascular disease, may have anti-inflammatory activities and therapeutic effects in diabetes, and also may act as an anticancer agent [64]. Okamoto et al. [65] also found that the fermentation water of *Lactobacillus plantarum* SN13T could strongly inhibit the release of IL-8, and sequencing revealed that the SN13T strain contained 11 genes encoding beta-glucosidase, which catalyzed the production of seco-tanapartholide C during fermentation to inhibit IL-8. Furthermore, pyruvate kinase (a specific pyruvate kinase M2 (PKM2) inhibitor that also inhibits TNF-α and NF-κB pathways) was detected. Based on previous studies and the results from this study, it can be concluded that *L. plantarum* Z22 may have anti-inflammatory potential. In addition, genes associated with antioxidant effects, such as thioredoxin reductase (TrxR), tyrosine-protein phosphatase, and ATP-dependent intracellular proteases ClpP, were determined. Zhang et al. [66] found that *Lactiplantibacillus plantarum* HOM3204, with strong antioxidant activity, can cope with oxidative stress; thioredoxin reductase related to antioxidants was also found during whole-genome sequencing. Liu et al. [67] found that the AGL003316 gene-encoded tyrosine-protein phosphatase in Lactobacillus plantarum DMDL 9010 plays an important role in regulating the synthesis of exopolysaccharide (EPS), while the EPS produced by probiotics has been proven to have antioxidant effects. Additionally, Zhang et al. [68] discovered that Lactobacillus plantarum ZLP001 encodes genes for some proteases involved in the oxidative stress response, such as ATP-dependent intracellular protease ClpP, which can protect proteins from abnormal damage. The appearance of these genes (Figure 5C) therefore provides a theoretical basis for our subsequent anti-inflammatory and antioxidant studies. In conclusion, *L. plantarum* Z22 is a promising candidate probiotic with potential future applications.

## 4. Conclusions

**Figure 6:**
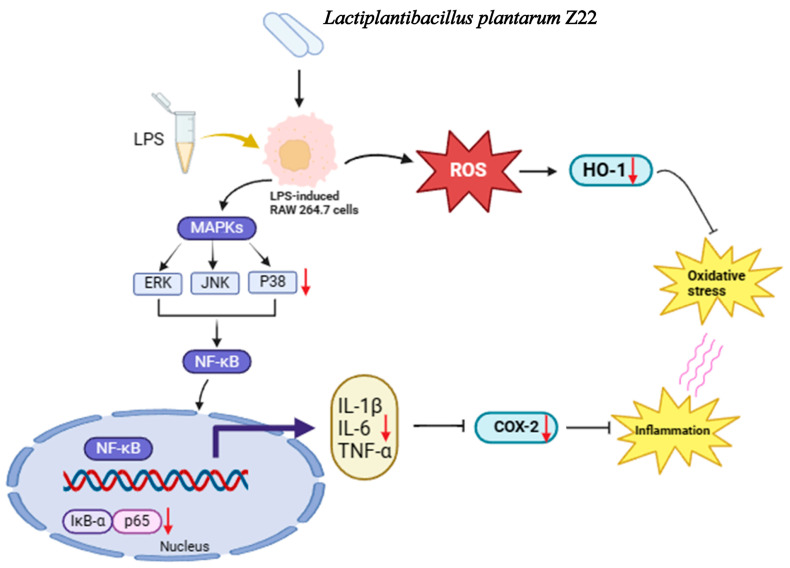
Proposed molecular mechanisms underlying the inhibitory effect of *L. plantarum* Z22 in LPS-stimulated RAW264.7 cells.

In this study, the probiotic properties and anti-inflammatory abilities of *L. plantarum* Z22 were explored. The results showed that *L. plantarum* Z22 has probiotic properties, such as tolerance to gastric acid and bile salts, adhesion capability, antimicrobial activity, and a favorable safety profile. In addition, this strain exerted its anti-inflammatory potential by inhibiting the activation of the NF-κB and MAPK signaling pathways, reducing the LPS-induced secretion of pro-inflammatory cytokines and COX-2 protein expression. This may be associated with its inhibition of ROS levels by upregulating HO-1 expression (Figure 6). Moreover, whole-genome sequencing revealed the presence of anti-inflammatory and anti-oxidation-related genes and the absence of active antibiotic resistance genes. In summary, the present study demonstrated that the anti-inflammatory potential of *L. plantarum* Z22 isolated from naturally fermented vegetables can be employed as an anti-inflammatory agent for the prevention and treatment of various inflammatory diseases, as well as for the development of nutritional supplements to meet the demand for anti-inflammation and immune enhancement. However, future research needs to explore its in vivo safety and efficacy to ensure further application.

## Figures and Tables

**Table 1:** Acid resistance and bile salt tolerance of *L. plantarum* Z22. Values are represented as mean ± SDs (*n* = 3). Data were considered as statistically significant when *p* < 0.05 (* *p* < 0.05).

Characteristics	Survival Rate (%) *
pH 2.5	102.20 ± 3.00
0.3% Bile Salt	101.19 ± 2.00

**Table 2:** Hydrophobic properties of *L. plantarum* Z22 and its adhesion to HT-29 cells. Values are represented as mean ± SDs (*n* = 3). Data were considered as statistically significant when *p* < 0.05 (* *p* < 0.05).

Characteristics	Survival Rate (%) *
Hydrophobicity	45.67 ± 3.06
Adhesion	72.75 ± 16.69

**Table 3:** Antimicrobial activities of neutral pH supernatants of *L. plantarum* Z22 against pathogenic strains. Add pathogenic bacteria to the LB agar plate (with a final concentration of 1 × 10^6^ CFU/mL), then add 100 µL of supernatant to the wells of the plate, incubate at 37 °C for 24 h, and measure the diameter of the inhibition zone with a vernier caliper. Values are accurate to 0.01 mm. Values are represented as mean ± SDs (*n* = 3). Means with different letters within a line indicate significantly different (*p* < 0.05). Data were considered as statistically significant when *p* < 0.05 (* *p* < 0.05).

Pathogen Strain	Inhibitory Zone Diameter (mm) *
*S. aureus* (ATCC 6538)	20.68 ± 0.03 ^a^
*E. coli* (CGMCC 9181)	19.27 ± 0.03 ^c^
*S. enterica* (ATCC 14028)	19.16 ± 0.04 ^b^

## Data Availability

The data are contained within this article.
